# Paying Patients at the Great Northern Central Hospital

**Published:** 1895-02-23

**Authors:** 


### HOSPITAL ADMINISTRATION.

[Questions and correspondence, marked "Hospital Administration" on the envelope, addressed to the Editor, are invited for this column.]

### PAYING PATIENTS AT THE GREAT NORTHERN CENTRAL HOSPITAL.

The annual meeting at the Great Northern Central Hospital, on Friday, cannot fail to have important bearings on the question of the admission of patients to metropolitan hospitals and the hospital system generally. At this meeting Dr. Glover will move—

That this meeting of governors of the Great Northern Central Hospital regret the recent changes in the administration of the hospital by which pay-patients have been admitted to its wards to be attended gratuitously by the honorary staff, and is of opinion that such a system is not likely to conduce to the welfare of the hospital or the advantage of the poor, for whom such institutions exist, and should without further delay be abandoned.

To which the following amendment will be moved by Mr. Burdett:—

That, with the two-fold object of loyally administering the affairs of the Great Northern Central Hospital on the lines of its constitution, and of reducing to a minimum the competition between the hospital and private practitioners, it is desirable that the system of pay wards now in operation be so modified that the patients in those wards may, if they desire it, be attended in the hospital by outside practitioners of their own selection; that the pay system, thus modified, be continued for an experimental period of five years; and that a committee of governors be appointed to prepare a detailed scheme on these lines, and to report to a meeting of the whole body of governors, to be convened for this special purpose in June next.

We have already discussed the whole question in our columns (*vide* The Hospital, November 18th, 1894, pages 91 and 92). We can only now hope that the Council of the Great Northern Hospital will fully realise that in coming to a decision in the case of their own hospital they will either give courage to those who desire the voluntary hospitals of this country to afford the maximum assistance to the public of all classes instead of being factors in a large scheme of pauperisation, or not only rob the hospital of some of the purpose of its existence, but weaken the cause of those who are struggling for enlightened administration.

